# Integrating genealogical and dynamical modelling to infer escape and reversion rates in HIV epitopes

**DOI:** 10.1098/rspb.2013.0696

**Published:** 2013-07-07

**Authors:** Duncan Palmer, John Frater, Rodney Phillips, Angela R. McLean, Gil McVean

**Affiliations:** 1 Department of Statistics, 1 South Parks Road, University of Oxford, Oxford OX1 3TG, UK; 2 Institute for Emerging Infections, The Oxford Martin School, Oxford OX1 3BD, UK; 3 Nuffield Department of Clinical Medicine, Peter Medawar Building for Pathogen Research, University of Oxford, Oxford OX1 3SY, UK; 4 Zoology Department, South Parks Road, University of Oxford, Oxford OX1 3PS, UK; 5 Wellcome Trust Centre for Human Genetics, Roosevelt Drive, Oxford OX3 7BN, UK

**Keywords:** phylodynamics, HIV, escape, genealogy, peeling, cytotoxic T-lymphocyte

## Abstract

The rates of escape and reversion in response to selection pressure arising from the host immune system, notably the cytotoxic T-lymphocyte (CTL) response, are key factors determining the evolution of HIV. Existing methods for estimating these parameters from cross-sectional population data using ordinary differential equations (ODEs) ignore information about the genealogy of sampled HIV sequences, which has the potential to cause systematic bias and overestimate certainty. Here, we describe an integrated approach, validated through extensive simulations, which combines genealogical inference and epidemiological modelling, to estimate rates of CTL escape and reversion in HIV epitopes. We show that there is substantial uncertainty about rates of viral escape and reversion from cross-sectional data, which arises from the inherent stochasticity in the evolutionary process. By application to empirical data, we find that point estimates of rates from a previously published ODE model and the integrated approach presented here are often similar, but can also differ several-fold depending on the structure of the genealogy. The model-based approach we apply provides a framework for the statistical analysis and hypothesis testing of escape and reversion in population data and highlights the need for longitudinal and denser cross-sectional sampling to enable accurate estimate of these key parameters.

## 1. Introduction

Cytotoxic T-lymphocytes (CTLs) are implicated in the control of human immunodeficiency virus 1 (HIV-1). In fact, they are thought to be the most important mediators in reducing viraemia in individuals able to control HIV infection, showing association with repression of viral replication in long-term non-progressors [1–3]. Epitopes are presented to CTLs by human leukocyte antigen (HLA) class I proteins at the surface of almost all nucleated cells in the body. The collection of epitopes which may be presented by the HLA class I molecules is determined by an individual's combination of alleles at these highly variable loci. Mutations in or close to epitopes in the viral sequence can result in alterations to the binding affinity to the HLA class I, reduce CTL recognition or abrogate T-cell receptor binding. Such mutations are known as escape mutations. Examples of escape mutations have been described in almost all proteins encoded in the HIV-1 genome [4–12], with the strongest signal of association with host HLA type at the HLA-B locus [13,14]. After an escape event takes place, escape mutations in the virus may be transmitted between individuals and thus have the potential to spread across the infected population [4,15], or revert through selection pressure within hosts whose immune responses do not drive escape in a given epitope [16,17]. Associations between HLA type and putative CTL escapes have been demonstrated statistically in population studies [18], though these results have been called into question [19], and it is suggested that the frequency at which escape events take place is lower than previously thought. More recent studies [20–22] have shown that there is a large variation in time to escape observed across epitopes, ranging from days to years. There is strong interest in understanding the selective pressures applied to the virus at the level of the population as there are clear implications for any putative vaccine. To date, simple ordinary differential equation (ODE)-based models have been used to estimate the expected time to escape and reversion by using cross-sectional data across hosts. Such estimates make use of only a small portion of the available data, namely presence or absence of an escape mutation and the HLA type of the sampled hosts (which we denote *E*), and disregard any remaining sequence information. These methods also make assumptions about the independence of the sampled data that could potentially lead to bias in estimates. Furthermore, deterministic models only provide point estimates and thus cannot provide meaningful confidence regions that account for phylogenetic uncertainty.

**Figure 1.**
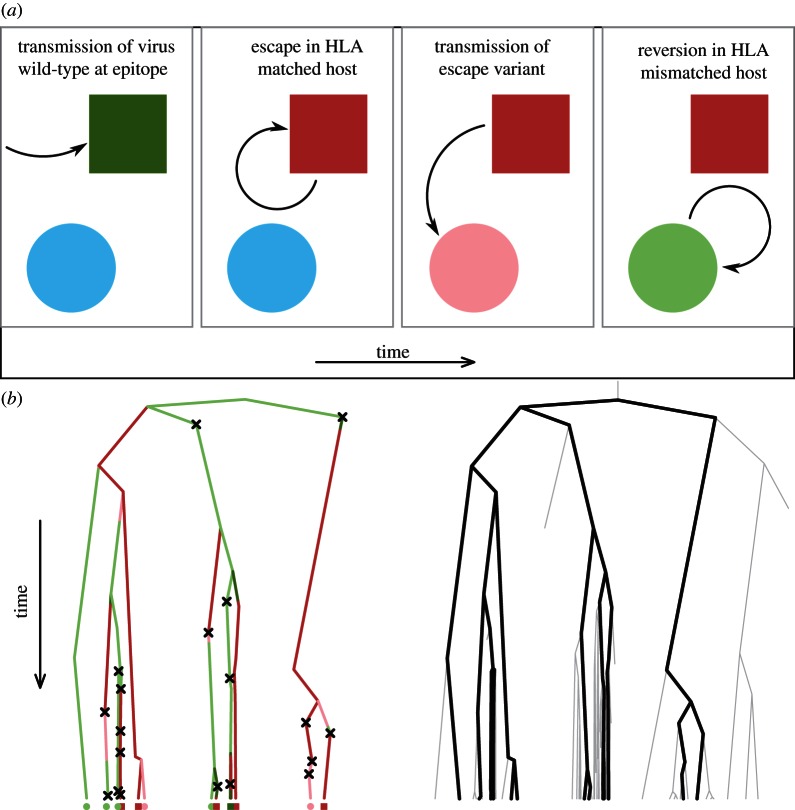
The inference problem. (*a*) The cartoon in displays the dynamic processes which may occur along a branch within the transmission tree. Time increases from left to right. A susceptible individual is shown in light blue. An individual infected with a virus which is wild-type at the epitope under consideration is green, and individuals with a viral strain with the escape mutation are red. HLA-matched hosts are darker rectangles, HLA-mismatched hosts are lighter circles. From left to right, transmission of a variant which is wild-type at the epitope under investigation occurs within the population. This strain may escape within an HLA-matched host. Transmission of an escaped viral strain to an individual who is HLA-mismatched can occur. This strain may then revert in this HLA-mismatched host. Viruses exist within these environments over their evolutionary history. Thus, associated to a collection of individuals sampled at the present (shown at the tips in (*b*)) is a colour-coded transmission tree, illustrated in (*b*). A transmission event is associated with each coalescence, but due to incomplete sampling, unseen transmission events also occur. These are shown by black crosses. This sampled transmission tree is embedded in the full transmission tree, shown in the second tree in grey. We have sequence data and colourings at the tips of a sampled transmission tree. Using these data, we hope to reconstruct the embedded tree in (*b*), and use this reconstruction to make inferences about the unknown full transmission process.

Figure 1 illustrates the inference problem that we are addressing. We wish to infer three rates: the rate of viral escape (switching from dark green to dark red in figure 1*a*), the rate of viral reversion (switching from pink to light green in figure 1*a*) and the transmission rate. If the underlying transmission tree was known, then the problem would be straightforward. However, we have only a collection of tip labellings (sequence data and HLA information) which are the culmination of an embedded subtree of the full process. To make statements about parameters of the full transmission tree, we must reconstruct the subtree together with the dynamic processes occurring along its lineages through time.

We apply dynamic programming in combination with existing software to combine phylogenetic and statistical approaches with well-studied, ODE-based modelling to integrate available sequence data. By combining these two frameworks we determine more informed estimates and credible regions of population-level escape and reversion rates, (*λ*_esc_, *λ*_rev_) which incorporate the underlying dependency structure present in the viral genealogy. There are four key steps in our inference:

— make the mild assumption that the genealogy, *G*, and HLA/escape information, *E*, are conditionally independent given the sequence information with the epitope removed; *X*;
— genealogies are sampled from the posterior conditional on *X* using the program Beast [23], with a coalescent prior for an exponentially growing population whose rate parameter is sampled in the Markov chain Monte Carlo (MCMC);
— for each sampled genealogy, we evaluate the posterior density for (*λ*_esc_, *λ*_rev_) using a modification of Felsenstein's peeling algorithm [24] based on the processes in figure 1*a*; and
— tree-specific posteriors are averaged to produce a final posterior density for (*λ*_esc_, *λ*_rev_).

We envisage similar methodologies being applied to a wide range of problems. Our model represents an addition to the highly active area of phylodynamics, in which both stochastic and deterministic approaches are being developed [25–27].

To test the robustness of our integrated method, we perform a series of simulations and compare the results with those of an existing ODE-based dynamical model [28]. Notably, our method does not require assumptions about the start time of the epidemic, or transmission rate during the exponential growth phase, as these are estimated by the model. When nucleotide substitution rates are fixed at estimates generated from empirical sequence data, we find that in simulation studies our model successfully estimates escape and reversion rates. By altering the nucleotide substitution rate, we find that a lack of information about the genealogy (through lower substitution rates) can dramatically affect escape and reversion rate estimations using our integrated approach, though we find that the rates of substitution found in HIV are sufficiently large for this effect to be considered negligible.

The integrated approach is then applied to estimate escape and reversion rates in four previously identified epitopes. The four epitopes were chosen in order to explore as much of the space of escape and reversion rates as possible on the basis of previous estimates generated using population-level data. Again, we compare our integrated method with the ODE method which generated these estimates [28]. We illustrate the benefit of setting our approach in a model-based framework by demonstrating some simple hypothesis tests.

Our model provides evidence for the hypothesis that rates of escape and reversion within host are slower than published estimates generated experimentally from individual case studies [20], and highlights the large amount of uncertainty inherent in estimates that make use of cross-sectional population data.

## 2. Methods

We wish to estimate escape and reversion rates within host, taking into account the dependency structure between sampled individuals arising from the phylogenetic tree. Throughout, we define hosts with an HLA type known to confer an escape mutation in the virus as HLA-matched, and those without such an HLA type as HLA-mismatched. We have the HLA types and cross-sectional viral sequences from a collection of hosts, taken from the Swiss–Spanish intermittent treatment trial (SSITT) [16,29]: a collection of 79, 67 and 53 HLA-typed sequences for the genes gag, pol and nef, respectively. Epitopes and associated HLA types are considered one at a time, independent of other epitopes. We consider four previously defined epitopes. These epitopes were chosen in order to test our method across as wide a range of escape and reversion rate parameter space as possible—based on previous rate estimates [28]. The chosen epitopes are shown in table 1 in column 1. Throughout, we abbreviate these epitopes by their first three amino acids (e.g. TST). By removing the epitope under consideration from sequences and determining the presence or lack of an escape mutation, we consider data from two processes. The sequence data with epitope removed, *X*, allow us to perform inference on the genealogy, *G*. The combination of HLA type and presence or lack of escape, *E* (which we refer to as HLA/escape information), provides information about the dynamical processes shown in figure 1*a*, which occur along the lineages of *G* over time. By assuming escape information is uninformative about *G*, *G* and *E* are conditionally independent given *X* (*P*(*G|X*, *E*) ≈ *P*(*G|X*)). This allows us to consider these two processes separately, with the second conditional on the first. Details are provided in the electronic supplementary material, §S1. By adding a collection of time-stamped data, taken from the Los Alamos HIV sequence database [31] to this HLA-typed cross-sectional sequence data, we create a DNA multiple sequence alignment [32] and perform some data-trimming. These time-stamped data are required for Beast to estimate a scaling from units of time measured in *generations* to *years*. After data-trimming, the resulting number of HLA-typed SSITT sequences are 55, 54 and 48 for gag, pol and nef, respectively. Given the alignment, genealogies are sampled using Beast with a standard coalescent prior for an exponentially growing population whose rate parameter is estimated in the MCMC. Taking a sample of genealogies from the Beast output, we consider the embedded tree for which we have HLA and escape information, and determine the likelihood of parameters (*λ*_esc_, *λ*_rev_) using a modification of Felsenstein's peeling algorithm [24]. Our peeling algorithm is based on the transitions shown in figure 1*a*. Defining 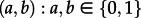, where 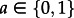 denotes {HLA mismatch, HLA match}, and 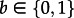 denotes {no escape mutation, escape mutation} in the epitope under investigation. In our model, transmission between individuals takes place at rate *λ*, individuals are assumed to be HLA-matched with probability *q*. Transitions between the states in the continuous time Markov chain may be described by the instantaneous rate matrix **Q** in equation (2.1), where *Q_i_*_,*j*_ describes the transition from state *i* to state *j*. 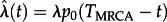, where *t* increases towards the present. *T*_MRCA_ is the time before the present of the most recent common ancestor (MRCA), where *t* = 0. *p*_0_(*t*) is the probability that a lineage at time *t* in the past does not have any sampled descendants [33], and depends on the sampling proportion at the present, *ρ*. Details of the derivation of *p*_0_(*t*) excluded from the original paper are provided in electronic supplementary material, §S2. The *p*_0_(*T* − *t*) scaling is required to avoid double counting of transmission events. We assume the state at the root node does not have the escape mutation. In our peeling algorithm, we must account for the fact that each internal node in the sampled genealogy corresponds to exactly one transmission event. For each sampled genealogy, we evaluate the two-dimensional posterior surface for the parameters of interest (*λ*_esc_, *λ*_rev_) using our peeling algorithm on a 50 × 50 lattice of parameter space that encompasses high probability density (within a factor of 1000 of the maximum density as determined by hill-climbing), conditional on *E* with a uniform prior over (10*^−^*^20^, 10^3^) × (10*^−^*^20^, 10^3^). We then integrate over 1000 samples from the Beast output to produce a final posterior density for (*λ*_esc_, *λ*_rev_), and define credible regions for our estimates. A description of the data and full details of the method are provided in electronic supplementary material, §S3.

**Table 1.** Summary of escape mutations analysed. Location is defined in parentheses by the HXB2 B-clade reference sequence. *q* is the HLA proportion in Caucasians [30]. Rates are in yr^–1^. Minimal and maximal rates of escape and reversion rate within the 95% credible regions for MAP estimates are in parentheses.

epitope	gene	HLA	escape	*q*	escape rate		reversion rate	
					MAP	ODE	MAP	ODE
TSTLQEQIGW	gag (108–117)	B57, B58	T3N	0.096	2.89 (0.138, 1000)	2.83	0.393 (0.0220, 3.40)	0.383
KRWIILGLNK	gag (131–140)	B27	R2K, R2G, R2Q	0.073	0.150 (0.00662, 2.66)	0.200	0.106 (0, 3.69)	0.154
TAFTIPSI	pol (128–135)	B51	I8T	0.126	0.194 (0.0274, 1000)	1.18	0.0281 (0, 0.242)	0.0562
RPMTYKAAV	nef (77–85)	B7	T4S, Y5F	0.166	0.0265 (6.06×10*^−^*^4^, 0.726)	0.0144	0.0385 (0, 1.09)	0.0226

As in ODE models of escape and reversion [28], we make a variety of assumptions. We assume homogeneous mixing in the infected population. We suppose that the infected population is in exponential growth and assume constant rates of escape in HLA-matched hosts, and constant rates of reversion in HLA-mismatched hosts. We ignore variation within individuals' viral populations. All escape mutations within a given epitope are assumed to occur at the same rate, and HLA types are considered to two digits. It is assumed that a single individual seeded the epidemic, and we suppose individuals with the corresponding HLA type are always able to make an immune response. Finally, recombination is not considered in our estimation of the genealogy, *G*.2.1
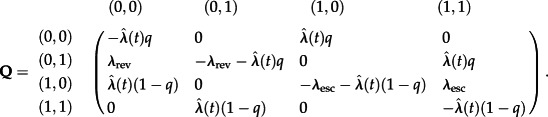


## 3. Results

### (a) Simulations

Under our model, assuming the population of infected individuals is in exponential growth, the process generating states at the leaves is a birth–death process [34] with escape and reversion events added. The birth rate is *λ*, and the death rate, *μ*, is equal to the rate of becoming non-infectious (through death or otherwise). Throughout our simulations, *q* = 0.15, *λ* = 0.45534 yr*^−^*^1^ (established from the average of a Beast run on gag data), *μ* = 0.1 yr*^−^*^1^. Where required, we sample 500 genealogies from Beast output. We find that this is sufficient due to the low variance in *maximum a posteriori* (MAP) estimates across sampled trees. This is due to uncertainty in the genealogy being concentrated at deep nodes, which have little impact upon the likelihood of observing data at the tips. We find the most recent coalescent events which have far more power to inform estimates are more consistent across sampled genealogies. There are three steps in our data simulation:

(1) Generate a full birth–death tree forwards through time with an MRCA of 25 years.
(2) Simulate HLA class I and escape information along lineages. Set the sampling proportion at the present, *ρ*, such that the expected number of present day tips is 200, and sample extinct tips at rate *ν* such that the expected number of historically sampled tips is 50. For step 3, we consider the embedded subtree defined by the sampling and discard HLA/escape information at historically sampled tips.
(3) Simulate sequence information at the tips, *X*, each 500 nucleotides long, using mutation parameters, *Θ* (set at the average of the required parameters from a Beast run on SSITT gag sequences) and a GTR + *I* + *Γ* model of substitution. Historical sequences are required by Beast to estimate a scaling from time measured in *generations* to *years*.

#### (i) Testing the integrated method and comparison with existing approach

**Figure 2.**
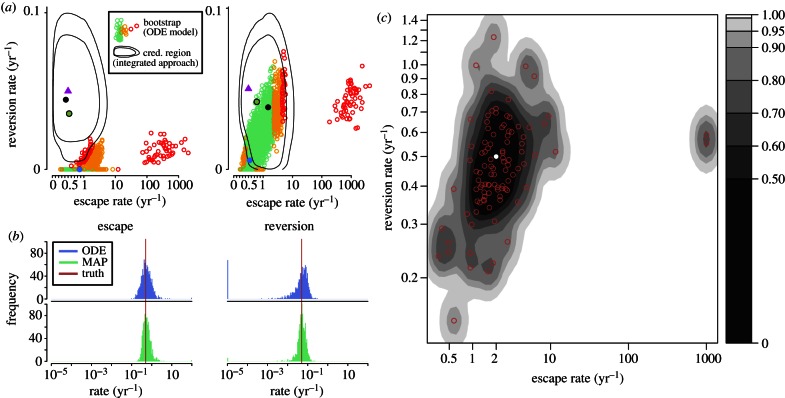
Simulation results. For each simulation, we generate a full birth–death process forward through time and set the sampling proportion; *ρ*, such that the expected number of sampled tips is 200. (*a*) Two instances of simulations under the integrated approach and the ODE model, the truth is set at (*λ*_esc_, *λ*_rev_) = (0.5, 0.05) yr*^−^*^1^. Estimates of 10 000 bootstraps of the data under the ODE model are shown as dots, coloured by Mahalanobis distance (the furthest 1% and 1–5% are coloured red and orange respectively, and the remainder green; see §2). The ODE point estimate based on the tip data is the blue dot. Ninety-five  per cent and 99% credible regions of the integrated method are in black. The MAP is the black dot. The MAP if the genealogy is known is the circled green dot. The purple triangle is the truth. Minimum and maximum escape and reversion rates in the 95% credible region are (0.0269, 3.14) and (0.0177, 0.0859) yr^–1^ respectively for the first plot, and (0.256, 6.21) and (1.90 × 10*^−^*^4^, 0.0834) yr*^−^*^1^ for the second. (*b*) MAP and ODE point estimates of 1000 simulations assuming the true tree is known. Histograms of ODE point estimates and MAP estimates are shown in blue and green, respectively. The truth is overlaid in red. The first and second columns are estimates of escape and reversion rates respectively. Rates less than 10*^−^*^5^ and more than 10^2^ are grouped. (*c*) The results of 100 simulations of the integrated model applied to data generated from underlying rates (*λ*_esc_, *λ*_rev_) = (2, 0.5) yr*^−^*^1^, shown as a white dot. 100 MAP estimates are shown in red. Contours are coloured using a two-dimensional kernel density estimate [36,37].

We test and compare our model with a differential equation approach previously described [28]. It can be shown that the dynamics of our model when the sampling proportion is zero match the relative proportions through time of the ODE model during exponential growth, and that this is equivalent to assuming a completely star-like genealogy in which all lineages emanate from the MRCA (see the electronic supplementary material, §S4). We perform three simulation studies:

(1) For each of the (*λ*_esc_, *λ*_rev_) parameter sets {(2,0.5), (0.5,0.5), (0.5,0.05), (0.05,0.05), (0.05,0.01)} yr^–1^, we apply steps 1–3. Using HLA/escape information at the present day tips, *E* and sequence information *X*, we apply the integrated method and ODE model. This process is repeated four times for each (*λ*_esc_, *λ*_rev_), and we compare the results. The ODE model requires an estimate of the transmission rate, death rate and the initiation time of the epidemic. We fix these parameters at their true values. We bootstrap *E* 10 000 times to provide an estimate of sampling uncertainty under the ODE model. Estimates of *μ* and *ρ* are required under the coalescent tree prior in an exponentially growing population, which we set at the truth. In order to define an approximation to confidence regions for sampling under the ODE method, we use the Mahalanobis distance measure [35] (see the electronic supplementary material, §S3) widely used in cluster analysis, which takes covariance between escape and reversion rates into consideration. Two instances of estimating the parameter set (0.5, 0.05) yr^–1^ are shown in figure 2*a*. Rate estimates based on the four simulations given the five parameter sets are shown in electronic supplementary material, figures S1–S5.
(2) We investigate further by running steps 1–2 1000 times for each of the parameter sets in step (1) comparing the MAP estimate under the integrated model on the true tree obtained in step 1 with the ODE point estimate. Results are shown in figure 2*b* and electronic supplementary material, §S8. We also check how both methods vary with the number of sampled tips by running this simulation on samples of size 60, 100, 200 and 500 in electronic supplementary material, figures S6–S9, respectively. This approximation to the full integrated approach is reasonable as we find point estimates for the MAP under the full method are similarly spread (e.g. compare figure 2*c* with electronic supplementary material, figure S8 row 1). The number of samples in the empirical data in the SSITT for the genes gag, pol and nef is 55, 54, and 48, respectively.
(3) We examine the ability of the integrated method to estimate true underlying parameters over a large number of simulations. Setting the truth at (*λ*_esc_, *λ*_rev_) = (2, 0.5) yr*^−^*^1^, we run steps 1–3 100 times and, for each, apply our full integrated method. Results are shown in figure 2*c*.

#### (ii) Robustness to tree topology and impact of mutation rate

In order to estimate the impact of uncertainty in the tree topology, we performed two tests. First, we run steps 1–2, permute tip labellings *E* in the true tree 250 times, re-estimating (*λ*_esc_, *λ*_rev_) each time using our peeling algorithm. Setting the true values at (*λ*_esc_, *λ*_rev_) = (0.6, 0.1) yr*^−^*^1^. (*λ*_esc_, *λ*_rev_) is set at an intermediate escape and reversion rate as such rates have the greatest amount of clustering in the true tree. Second, we apply steps 1–3, but multiply each substitution rate in step 3 by a factor of 5 and 0.2, before applying the integrated method. The truth is set at (*λ*_esc_, *λ*_rev_) = (2, 0.5) yr*^−^*^1^.

### (b) Simulation results

#### (i) Simulations with known underlying tree (simulation study (2))

By determining MAP of 1000 instances of HLA and escape data, supposing we know the true underlying genealogy, we find our integrated method best estimates the true rates when the truth lies in the centre of our range of parameter simulations. Results are shown in figure 2*b* and electronic supplementary material, figure S8. The proportion of MAP estimates within a factor of 2 of the truth was {0.771, 0.825, 0.809, 0.468, 0.268} for parameter sets (*λ*_esc_, *λ*_rev_) = (2, 0.5), (0.5, 0.5), (0.5, 0.05), (0.05, 0.05), (0.05, 0.01) yr^−1^. This makes sense: very high or low escape leads to a lack information to discern from either an infinite rate, or a rate of 0. Such estimates will result by chance under non-zero rates, the extreme example is data in which all individuals show escape. Our integrated approach substantially outperforms the ODE method when escape and reversion rates are slow. As we increase the number of sampled tips in genealogies (see the electronic supplementary material, figures S6–S9, sampling 60, 100, 200 and 500 tips, respectively), we see increased accuracy under both methods, most notable at fast and slow rates. We find that the integrated method consistently outperforms the ODE method, even when the number of sampled tips is low.

#### (ii) Simulations with unknown underlying tree (simulation studies (1) and (2))

We conduct four simulations over the five parameter sets of escape and reversion rates (*λ*_esc_, *λ*_rev_) = (2, 0.5), (0.5, 0.5), (0.5, 0.05), (0.05, 0.05), (0.05, 0.01) yr^–1^ shown in figure 2*a* and electronic supplementary material, §§S1–S5, and a further 100 simulations for (2,0.5) yr^–1^ shown in figure 2*c*. We find large variation in the size of credible regions across genealogies, particularly for low underlying rates. In the large simulation shown in figure 2*c*, true parameters lie within the 50, 90 and 95 per cent credible regions 79, 92 and 95 times of 100.

#### (iii) Comparison with ordinary differential equation method

We find that in general, the ODE method estimates the truth well, particularly when escape and reversion rates are fast. This makes sense: along branches culminating in tips, fast escape and reversion leads to convergence to the equilibrium distribution, which is independent of the tree. For slower rates however, our integrated method is favourable. This can be seen in single simulation runs in figure 2*a* and electronic supplementary material, §§S1–S5, and in rate estimates assuming the true tree is known (figure 2*b* and electronic supplementary material, figure S8). Here, the integrated approach performs far more favourably, with a tighter distribution about the truth in all parameter sets. However, as would be expected, the signal begins to drop under both models as underlying rates are reduced further. For the underlying parameter sets {(2,0.5), (0.5,0.5), (0.5,0.05), (0.05,0.05), (0.05,0.01)}, the proportion of ODE point estimates and MAP estimates within a factor of 2 were {0.768, 0.823, 0.570, 0.214, 0.055} and {0.771, 0.825, 0.809, 0.468, 0.268}, respectively (the corresponding values within a factor of 10 were {0.982, 0.999, 0.913, 0.679, 0.356} and {0.982, 0.999, 0.990, 0.850, 0.515}, respectively). Knowledge of the transmission tree increases accuracy of rate estimations across the parameter space of (*λ*_esc_, *λ*_rev_), particularly when escape and reversion rates is low.

#### (iv) Robustness to tree topology and impact of mutation rate

By shuffling tip labellings, we investigate robustness of estimates to the tree topology. We find, in addition to an increase in the variance of estimations, a systematic bias towards higher rate estimates, shown in electronic supplementary material, figure S10. This makes intuitive sense: reduction in knowledge of tip labellings will act to randomize any clustering (or lack of clustering) present in the true tree of escaped and wild-type strains at the epitope under consideration, leading to a forced increase (decrease) in the lower bound of the number of escape and reversion events in the tree, increasing (reducing) rate estimates. To investigate the effect of mutation rate on estimates, we multiply and divide substitution rates by a factor of 5. This is displayed in electronic supplementary material, figure S12. As the mutation rate is increased, we see a reduction in the variance and increase in the accuracy of estimates as we would expect. It is important that this observation is considered in pathogens in which mutation rates are far lower and phylodynamic methods are beginning to be applied. Electronic supplementary material, figures S10 and S11 demonstrate that a lack of knowledge of the underlying genealogy can seriously impact any parameter estimations leading to potentially spurious results.

### (c) Analysis of real data

**Figure 3.**
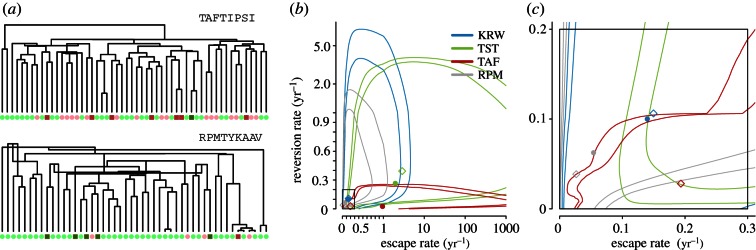
(*a*) The maximum clade credibility tree for cross-sectional data for epitopes TAFTIPSI and RPMTYKAAV, with tips coloured as in figure 1. (*b,c*) 95% and 99% credible regions under our integrated method. (*c*) A zoom in of the rectangle in (*b*). Axes are linear on [0,1] and on a log scale for values more than 1. Colourings are shown in upper right figure legend of (*b*). Coloured diamonds are MAP estimates of (*λ*_esc_, *λ*_rev_), ODE method estimates are filled circles.

The result of applying the integrated method to the available SSITT data is displayed in figure 3 and summarized in table 1. The cross-sectional proportion sampled, *ρ*, is set at 0.003, based on incidence data [38]. The first aspect to note, which was also present in many of our simulations, is the similarity between the simple ODE method and the MAP from the integrated approach. This is not surprising as the purely dynamical model [28] can be written as a composite likelihood, which results from the assumption that all lineages are independent and of equal weight. The success of composite likelihoods is reflected in the similarity seen. However, looking more closely at the TAF and RPM epitopes (table 1), we see that the estimate of the underlying genealogy is playing a strong role. The maximum clade credibility trees for the Beast runs of TAF and RPM using TreeAnnotator [23] are shown in figure 3*a*. We see that in the case of TAF, the escape rate is approximately six times lower in the MAP estimate than the ODE estimate. This is reflected in the clustering seen in the tree. Of the 24 individuals who have a consensus escape, 13 occur in clusters of two or more. Moreover, singleton-escaped lineages coalesce deep into the tree. These combine to reveal the existence of a lower escape rate than that seen in the ODE approximation, combined with transmission of escapes indicative of a very low reversion rate. Contrast this to the rate approximation in RPM. Recent coalescent events in which one lineage has the escape variant, whereas the other does not leads to faster escape and reversion estimates under the integrated approach. Second, we note the large amount of uncertainty arising through the evolutionary process in our estimations. This can be explained through two main factors: the lack of seen transmissions close to the present, and uncertainty in the states deep into the ancestry. Improvement in each will result from increased sampling. An increase at the present will increase the number of recent coalescent events, and increased historical sampling will add confidence to inferred states deep in the genealogy. Throughout, we have considered the coalescent in an exponentially growing population as our prior on the genealogy, as a birth–death prior with historical sampling and sampling at the present is currently untested in Beast. Use of a birth–death prior would increase uncertainty in the time to the MRCA owing to its stochastic nature. Indeed, the deterministic nature of the coalescent underestimates uncertainty in *T*_MRCA_ in an epidemic setting. However, using a sampled birth–death prior allows us to sample large portions of the infected population completely correctly, increasing our power to infer dynamical parameters. Unfortunately, the long-terminal branches indicative of exponential growth means that it will always be difficult to estimate parameters of such dynamical models.

Major advantages of the model-based framework are that we obtain meaningful credible intervals for our parameter estimates, and gain a statistical framework in which hypotheses about these parameters can be tested. For example, consider the null hypothesis that escape and reversion rates are common across the four epitopes, with the alternative that they each have distinct rates. Using a likelihood-ratio test, we reject the null hypothesis (*p* = 0.00014). Testing the difference in escape and reversion rates between RPM and KRW, we find the data cannot reject the null hypothesis that there is a common escape and reversion rate across these two epitopes (*p* = 0.412). Validation of our use of the likelihood-ratio test is given in electronic supplementary material, §S5.

## 4. Discussion

We have combined a dynamical modelling approach with cross-sectional sequence data to infer escape and reversion rates at CTL epitopes within hosts while taking the underlying genealogy into account. We compared our integrated approach with this ODE-based method through simulations and parameter estimates using cross-sectional data from the SSITT study cohort. Our model is set in a statistical framework and makes use of the information present in sequence data for parameter estimates. We gain meaningful credible regions which consider uncertainty in the true underlying genealogy. A strong benefit of our integrated method is that it provides a probabilistic framework in which hypotheses can be tested. Our most striking conclusion is the large amount of uncertainty present in rates estimates using cross-sectional sequence data. Great care must therefore be taken before strong conclusions are made on the basis of such estimations.

Under the ODE approach outlined in Fryer *et al*. [28], sequence information outside the epitope under consideration is redundant. Cross-sectional data are considered to have arisen independently, up to initial conditions. We show that this is equivalent to the assumption of a completely star-like phylogeny in electronic supplementary material, §S4. Given this major assumption of the ODE method, we expected our integrated method to perform more accurately in simulations. We find that this is indeed the case, the presented model consistently outperforms the simpler ODE-based approach, particularly when strong clustering of escape mutations is more likely to be present in the true tree. Despite this improvement, we find that the ODE method recaptures escape and reversion rates relatively well in simulations, in spite of its simplifying assumptions.

In order to incorporate the underlying dependency structure present in the genealogy of our samples, and assuming exponential growth, there are two major processes to decide between: the coalescent in an exponentially growing population [39–41] (which we chose) and the sampled birth–death process [33,42]. In the case of the birth–death process, a large number of likelihoods describing the process have been constructed [33,43–45], differing only in what the author(s) decide to condition on. We summarize the links between each likelihood in electronic supplementary material, §S6. Our choice between exponential coalescent and sampled birth–death process determines the prior on the genealogy. One major distinction is that the sampled birth–death process considers a subpopulation within a larger stochastically growing population, and the exponential coalescent assumes that the subpopulation is a tiny subset within a very large deterministically growing population. There are benefits and drawbacks to each. Under a birth–death prior, the sum of seen transmissions informed by the prior and unseen transmissions given by the matrix **Q** is the overall transmission rate over time—a desirable property which the coalescent lacks [42]. The birth–death process incorporates early stochasticity, which more accurately represents the truth in an epidemic setting. Additionally, no coalescent assumption is required, making the prior suitable for datasets in which the number of samples is comparable to the total population size. This is becoming increasingly relevant as such datasets are becoming more commonplace [46]. Under the exponential coalescent, inclusion of time-stamped datasets is straightforward. By contrast, under the birth–death process, assumptions about the sampling rate of these historical events must be made [47], which are often invalid for many datasets. However, both are only priors on tree shape and if the data are strong, then the distribution from which the trees are sampled will be near identical. We show an interesting link between the two processes in electronic supplementary material, §S7.

In our estimates, we are fundamentally restricted by coalescence events in the genealogy. Long-terminal branches are indicative of exponential growth, yet the greatest power to inform our parameter estimates comes from coalescence events occurring in the recent history of the virus. Thus, obtaining extra information from the genealogy is intrinsically difficult. Greater sampling at the present will increase the occurrence of recent coalescence events, and provide greater power to distinguish high and low rates from infinity and zero, respectively. However, the inclusion of more and more sequence data calls the coalescent assumption into question. Dense sampling can also lead to a breakdown of the assumed connection between the genealogy and the transmission tree owing to lineage sorting [48]. Including time-stamped data with tip information would allow estimation of ancestral states with greater confidence, and thus increase our ability to infer escape and reversion rates. If longitudinal and cross-sectional sequence data could be combined, this would dramatically increase power to estimate rates. Unfortunately, current methods cannot support this extension due to the use of the genealogy as a proxy for the transmission tree. It is clear that greatest power to estimate these parameters lies in longitudinal sampling within cohorts of hosts, but here we create another collection of issues: recombination plays a large role within hosts [49], and we would require longitudinal sequences across a large number of individuals in order to make any meaningful statements about rate estimates across the infected population.

Models that attempt to integrate the underlying genealogy are currently being developed to incorporate epidemiological dynamics outside the exponential growth phase [50] assumed under this model. Another potential improvement would be to co-estimate escape and reversion rates within the MCMC scheme. Our model also makes many assumptions about the underlying biological processes. For example, overlapping epitopes which are prevalent across the HIV genome [51] mean that mutations conferred by one HLA type could be incorrectly inferred to be the result of selection due to another HLA type. Violation of this assumption of selectively neutral sites outside the epitope of interest will affect branch lengths more than topology, so should not greatly alter our estimates and their ordering within genes. It is through changes in topology in the recent history of the data that estimates will be most drastically altered. HLA types are considered to two digits and escape mutations within a given epitope are grouped together owing to the relatively small dataset. All individuals with the restricting HLA type are assumed to be capable of making a response which drives selection at the epitope under investigation. With larger datasets, such complications could be included in a similar model in the same framework with more parameters. Throughout, we assume homogeneous mixing. This assumption may affect our estimates and will have the greatest impact if the HLA distributions are strongly segregated across the host population. Considering individuals from across continents, for example, would be highly inappropriate. HLA distributions across Europe are relatively homogeneous, so we feel that this assumption is reasonable. A variety of other hypotheses could be tested by extending the model in the presence of extra data. For example, given disease outcome information, HLA typing and sequence information, is it possible to discern that escape mutations are associated with faster disease progression?

We have constructed a model that integrates sequence data and considers the evolutionary history, transmission and set of dynamical processes together. The model was created using existing techniques, and we use it to address pressing practical questions. While we created the presented model to answer a specific question, we believe that similar integrated models making use of epidemiological and viral sequence data may be more broadly applied. Such models can be used to estimate various parameters of interest more accurately with the help of sequence data.
